# NURSE PRACTITIONER–LED IMPLEMENTATION OF HUDDLES TO SUPPORT STAFF IN LONG-TERM CARE HOMES

**DOI:** 10.1093/geroni/igac059.2039

**Published:** 2022-12-20

**Authors:** Katherine McGilton, Alexandra Krassikova, Aria Wills, Margaret Keatings, Jennifer Bethell, Veronique Boscart, Souraya Sidani

**Affiliations:** KITE Research Institute: Toronto Rehabilitation Institute-UHN, Toronto, Ontario, Canada; Toronto Rehabilitation Institute, University Health Network, Thornhill, Ontario, Canada; Toronto Rehabilitation Institute, Toronto, Ontario, Canada; Toronto Rehabilitation Institute, Toronto, Ontario, Canada; Toronto Rehabilitation Institute, Toronto, Ontario, Canada; Conestoga, Kitchener, Ontario, Canada; Ryerson University, Toronto, Ontario, Canada

## Abstract

Staff working in long-term care (LTC) homes frequently report experiencing moral distress related to lack of autonomy and not being able to provide quality care. Huddles have been used as a communication tool for many years in acute care settings to improve collaboration and safety culture. In LTC homes, huddles are implemented less often, despite evidence of their benefits in improving support and teamwork. In this pre-test post-test implementation study, huddles led by a nurse practitioner (NP) were introduced in a privately-owned not-for-profit LTC home with < 150 beds, located in a medium urban centre in Ontario, Canada. Objectives of the study were to 1) examine fidelity of huddle implementation; 2) examine the extent to which the huddles improved staff’ outcomes of moral distress, job satisfaction, and support provided by the NP estimated with Bayesian proportional odds model. A total of 48 huddles were carried out by the NP over 15 weeks. Huddles were most commonly attended by personal support workers (98%) and registered practical nurses (96%), with an average of 7 individuals per huddle. Topics most often addressed at huddles were related to resident care (46%) and staff concerns (34%). Strong statistical evidence of a reduction in overall moral distress was evident for staff attending the huddles, when compared to staff who did not (posterior probability =.9933). No changes in job satisfaction and support provided by the NP were observed. Introducing huddles in LTC homes may be effective at reducing moral distress experienced by staff.

